# Lifetime cost analysis of concrete barriers and steel guardrails

**DOI:** 10.1038/s41598-024-66090-1

**Published:** 2024-07-08

**Authors:** Alireza Nemati, Meridian Haas, David Torick, Shima Nazari

**Affiliations:** Department of Mechanical and Aerospace Engineering, University of California-Davis, Davis, CA USA

**Keywords:** Lifetime cost analysis, Roadside barriers, Concrete barriers, Steel guardrails, Civil engineering, Mechanical engineering

## Abstract

This study investigates the lifetime costs associated with concrete barriers and steel guardrails. We introduce a cost analysis methodology that incorporates critical factors such as construction costs, maintenance costs, exposure risks during maintenance activities, and the costs imposed to traveling public through the increased traffic and the crash outcomes. We integrate various parameters including economic factors, road geometry, general weather condition, and traffic mix to estimate a location-dependent cost for each type of barrier accurately. A software tool, named CalBarrier, was developed during this study to carry out the calculations and the comparison of lifetime cost of aforementioned barriers. An inherent strength of this research is its reliance on recent real data extracted from various databases of California Department of Transportation (Caltrans), ensuring precision and relevance in accounting for various influential factors. Drawing insights from Caltrans practices and interviews with their personnel, this study emphasizes the intricate decision-making process involved in mitigating safety risks and reducing operational expenses. Although our data originates from California, the methodology for life cycle cost analysis, and our software are applicable for regions with different socio-economic conditions by deploying user input costs, making our findings a valuable resource for other areas facing comparable challenges.

## Introduction

Roadside barriers play a critical role in increasing the safety of highways by mitigating potential risks for road users. These barriers come in various forms and sizes, including concrete, steel, and cable barriers. Each type is designed in multiple sizes and optimized to withstand a specific impact capacity. While all three types can be observed on roads, concrete and steel barriers emerge as the most prevalent. Selecting the appropriate type and size demands thorough consideration, prompting the need for careful studies. Several guidelines and standards have been established to assist road designers in choosing suitable barriers that meet specific requirements. In the United States, the American Association of State Highway and Transportation Officials (AASHTO) has published the Highway Safety Manual (HSM), providing tools for quantitative safety analyses on existing or proposed roadway^1^. The Manual for Assessing Safety Hardware (MASH) offers uniform guidelines for crash testing of highway safety features, along with evaluation criteria for interpreting test results^2^. Various regional organizations have published their own guidelines to aid designers in selecting appropriate roadside barriers. Notably, the California Department of Transportation (Caltrans) released the latest version of the Highway Design Manual (HDM) in 2019^3^, encompassing a wide range of aspects, from the application of design standards and basic policies to intricate details of highway design.

The selection of a suitable roadside barrier has traditionally been dominated by specified requirements. However, in recent decades the emphasis has shifted towards identifying the most cost-effective option. Although steel guardrails boast a substantially lower initial construction cost, concrete barriers offer significant advantages that make them preferable in various conditions. The total ownership cost of a barrier is contingent on numerous parameters, which vary across countries and regions. Disparities in labor and material costs can significantly impact the expenses related to construction and maintenance of barriers. Therefore, many cost–benefit analyses of the barrier have been conducted locally and internally by many organizations and companies, and only a limited number of studies have been published regarding computing the lifetime cost and conducting cost–benefit analyses of roadside barriers.

In 2012, Karim et al.^4^ developed an approach for analyzing the life-cycle cost (LCC) of barriers, incorporating construction and maintenance costs in their LCC study. Alipour et al.^5^ investigated the LCC of concrete bridge barriers exposed to chloride. William et al.^6^ conducted a cost–benefit analysis of median barriers, focusing on injuries and the cost of vehicle crashes into barriers. The inclusion of costs imposed on road users due to traffic delays resulting from crashes into barriers is a key aspect of a comprehensive LCC study. Karim et al.^7,8^ assessed the injury rate associated with roadside barriers. Several studies and guidelines have been published with the aim of estimating traffic delays resulting from lane closures during maintenance activities^9–11^. Tufuor et al.^12^ estimated travelers' costs due to lane closures caused by maintenance and construction activities. In prior studies, construction and maintenance costs were estimated based on data published several years earlier than the study and sourced from various regions^13,14^. These studies often focused on a single type of barrier, neglecting the impact of road geometry, traffic mix, and economic factors on the lifetime cost of barriers. Moreover, the existing literature encompass only a few factors in their cost–benefit analysis and fail to provide a comprehensive analysis including all key factors.

**Figure 1:**
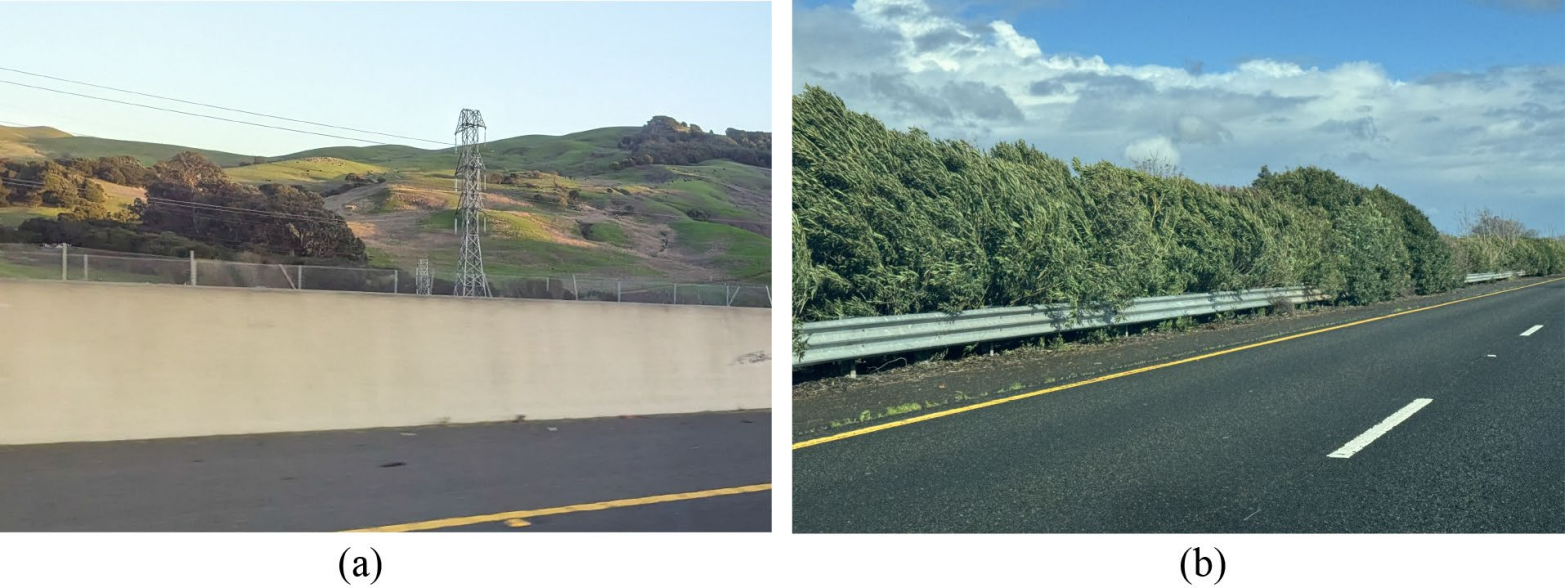
Samples of typical median barriers installed on highways I-80 near Sacramento, California, USA. (**a**) Concrete barrier. (**b**) Thrie-beam steel barrier. Vegetation management is required around the steel barrier.

This study provides an extensive lifetime cost analysis of concrete and steel barriers, the two most common types on highways, shown in Fig. 1. Our study incorporates construction costs, maintenance expenses, and costs imposed on the traveling public through roadside barrier type. Moreover, we consider indirect costs associated with exposing workers and equipment to traffic during maintenance activities. Additional indirect costs related to specific barriers, such as preventing vegetation growth around guardrails, are included in this life-cycle cost study. Importantly, we utilize the most recent data extracted from various Caltrans databases to compute real-world construction and maintenance costs of barriers. As highlighted earlier, costs may vary in different countries and regions. Nevertheless, this study introduces an approach to LCC analysis applicable in any region. It should be noted that the research outcomes of this study are directly applicable to regions with socioeconomic conditions similar to California, USA. Additionally, a publicly available and free software, developed as part of this research facilitates the computation and comparison of LCC for various barriers, considering factors such as traffic mix, road geometry, and economic conditions. Incorporating the feedback from a series of interviews with Caltrans personnel responsible for barrier design, maintenance, and construction, adds another layer of value to this research. Finally, we list some indirect environmental impacts of barriers, acknowledging the inherent challenges in quantifying these impacts.

The contributions of this study are multi-faceted. First, we present new equations derived from extensive data analysis and theoretical modeling, which accurately estimate the lifetime costs of barriers by considering road geometry, traffic mix, and economic factors. Second, we introduce a data-driven approach to quantify crash costs, incorporating a regression model based on high-fidelity simulation data. Third, we identify and quantify indirect costs such as worker exposure risks and public costs due to traffic delays, which are often overlooked in traditional cost analyses. Finally, our publicly available software, CalBarrier, provides a practical tool for applying our methodology in diverse regions, ensuring the transferability and applicability of our findings.

The rest of the paper is structured as follows: Section "LCC methodology" presents the methodology used in this study to compute various related costs. Section "Results and discussion" lists the maintenance and construction costs of various types of barriers. The cost of workers and equipment exposure to high-speed traffic during maintenance activities is also detailed in this part. Section "Cost imposed on public" focuses on the costs associated with traffic delays and vehicle collisions with barriers. Section "The environmental considerations" discusses environmental considerations, and Section "CalBarrier" introduces CalBarrier, the software developed to compute the LCC of barriers. Finally, Sect. 7 presents a conclusion of the research outcomes.

## LCC Methodology

In this study, the lifetime cost of the barriers was calculated across four categories: construction, maintenance, exposure, and public cost.

1 $$LCC=CC+MC+EC+PC$$

in Eq. (1), *LCC* is the lifetime cost of the barrier, *CC* is the construction cost, *MC* is the maintenance cost, *EC* is the cost of workers' exposure to traffic during maintenance, and *PC* is the public cost. The construction cost can include the installation of a rubber mat or concrete pad to prevent vegetation growth near the barrier if necessary. The public cost consists of two categories: the cost imposed on the public resulting from crashes into the barrier, and the cost of traffic delays during the maintenance of the barriers. The maintenance cost of the barriers includes the regularly required maintenance as well as necessary repairs due to crashes to the barriers and weather conditions. Replacement of the damaged barriers is included as a part of the maintenance; therefore, no extra cost was considered for end-of-life removal in this project. Nevertheless, it should be mentioned that the removal cost of healthy barriers due to any other reasons such as upgrading and widening the highways is out of the scope of this study. All the costs associated with the barrier during its lifetime in future years, including maintenance, exposure, and public costs, will be adjusted according to the inflation rate. The computed cost is also converted to its current value by considering the interest rate utilizing the *present value of a future cost,* more details about this method are provided in^15^*.* Equation (2) was employed to calculate the present value of a future cost in this study.

2 $$PV=\frac{FV}{{(1+r)}^{n}}$$

where $PV$ represents the present value of the future cost, $FV$ is the future value or the cost in the future, $r$ is the discount rate, representing the rate at which money is discounted over time, and $n$ is the number of time periods until the future cost is incurred. Future costs are adjusted based on the inflation rate, as described in Eq. (3)

3 $$FV=CV{(1+i)}^{n}$$

in which $i$ is the inflation rate, and $CV$ is the cost computed in 2023. The raw data were extracted from the Caltrans Contract Cost database, and the Caltrans Integrated Maintenance Management System (IMMS) covering the period from 2020 to 2022, which included more than 150,000 work orders^16^. The construction, maintenance, and exposure costs were computed using the extracted data, and were adjusted for inflation according to the inflation rate of each year.

## Results and discussion

### Initial cost

Using the data obtained from the Caltrans Contract Cost database, we computed the initial construction cost for each type of barrier per unit of length. The results are summarized in Table 1.

**Table 1:** Mean construction cost of various types of barriers, acquired from Caltrans Contract Cost database for 2020–2022 period.

Barrier type	Unit	Cost
Concrete Barrier	Foot	$105.14
Thrie-Beam Barrier	Foot	$44.36
W-Beam Barrier	Foot	$30.94

The results presented in Table 2 represent the weighted average costs of concrete barriers constructed in California over the 2020 to 2022 period in several different forms and sizes.

**Table 2:** Average construction cost of various types of concrete barrier calculated using the Caltrans Contract Cost database for 2020–2022 period.

Barrier type	Unit	Cost
Type 60M	Foot	$84.31
Type 60MC	Foot	$115.60
Type 60MD	Foot	$96.17
Type 60MF	Foot	$304.94
Type 60MG	Foot	$97.15
Type 60MFG	Foot	$288.38
Type 60MS	Foot	$94.09

It's worth mentioning that some types of concrete barriers, such as Type 60MFG and Type 60MF, have significantly higher construction costs compared to other concrete barriers. However, they have relatively limited usage and are only used when the road geometry necessitates their usage. Table 3 provides detailed information on the construction costs per unit of length for various types of steel barriers.

**Table 3:** Average construction cost of various types of W-beam steel barrier computed from the Caltrans Contract Database for 2020–2022 period.

Barrier Type	Unit	Cost
With 6’ Steel Post	Foot	$33.82
With 6’ Wooden Post	Foot	$31.76
With 7’ Steel Post	Foot	$36.62
With 7’ Wooden Post	Foot	$35.92
With 8’ Steel Post	Foot	$43.17
With 8’ Wooden Post	Foot	$39.30

Wooden guardrail posts are common in some districts of California. Although wooden guardrail posts are cheaper than their steel counterparts, they don't substantially change the initial cost of the barrier due to their higher installation cost.

#### Vegetation management cost

It is estimated by Caltrans that approximately 50–60% of California roads require some type of vegetation management around guardrails. Other areas do not need vegetation growth prevention due to their weather conditions. Two methods are commonly used for vegetation management: covering the ground with concrete or using rubber (fiber) mats. In the case of concrete barriers, vegetation management is not usually necessary because concrete is poured up to the pavement, and the growth of undesirable vegetation is unlikely.

According to the Caltrans Contract Cost database, the average cost of a vegetation control mat (rubber or fiber) was $57.96/SQYD, and on average, Caltrans ordered 46,987 square yards per year^16^. On the other hand, the average cost of concrete covering is around $60.94/SQYD, which is slightly higher than rubber mats. Nevertheless, on average, Caltrans ordered around 822,925 square yards of concrete covering per year during the 2020–2022 interval. Rubber (or fiber) mats are less expensive and simpler to install than covering the surface with concrete. However, rubber and plastic mats are vulnerable to fire, so they are not usually used in fire-prone areas. The extracted data showed that despite the cheaper cost, rubber mats have been used far less compared to concrete covering. The additional cost of vegetation management around steel barriers averaged $20.31 per linear foot of barrier during the 2020 to 2022 interval.

In situations where it is deemed necessary to manage vegetation growth around the barriers, the cost of vegetation management is added to the overall construction cost as presented in Eqs. (4) and (5). ${C}_{C}$ and ${C}_{s}$ represent construction cost of concrete and steel barrier, respectively, and *V* shows the cost of vegetation management (if necessary). The cost associated with covering the ground to prevent vegetation growth is the same for both types of considered steel guardrails.

4 $${C}_{C}={BCP}_{C}.L.\frac{{(1+i)}^{(Y-2023)}}{{(1+r)}^{(Y-2023)}}$$

5 $${C}_{s}={(BCP}_{s}+V).L.\frac{{(1+i)}^{(Y-2023)}}{{(1+r)}^{(Y-2023)}}$$

where $${BCP}_{c}$$ is the base construction cost of concrete barrier listed in Table 2. $${BCP}_{s}$$ is the base construction cost of steel barrier listed in Table 3. $$V$$ is the base vegetation control cost which is equal to $20.31/ft. $$L$$ is the road length, $$i$$ is the inflation rate, $$r$$ is the interest rate (nominal discount rate), and $$Y$$ is the construction year of the project.

### Maintenance cost

The average cost of maintenance and repair of barriers from 2020 to 2022 in the state of California has been compiled and is presented in Table 4. The repair costs have been computed using the Caltrans IMMS database. The results demonstrate that the maintenance cost of concrete barriers is considerably lower compared to their steel counterparts.

**Table 4:** Average cost of maintenance and repair of different types of barriers computed using data from the Caltrans IMMS database for 2020–2022 interval.

Barrier type	Unit	Cost
Concrete Barrier	per foot per year	$0.075
Thrie-Beam Barrier	per foot per year	$1.01
W-Beam Barrier	per foot per year	$1.77

The characteristics of a road exert a significant influence on the frequency of crashes, thereby impacting the repair costs of barriers. In this work we introduce a method to account for the impact of road geometry and other characteristics on the maintenance costs of a segment of the road by introducing a modification based on encroachment rate. Our approach assumes that a higher encroachment rate translates into more barrier collisions and thereby a higher maintenance cost. The introduced modification factor, ${A}_{enc}$, is computed utilizing the analytical method presented by Carrigan et al.^17,18^ and the RSAPv3 method^19^, which provide a set of tables for the modification of encroachment rate based on road geometry. Note that the prior literature use encroachment rate to estimate the crash costs for the traveling public; in this work, for the first time, we adopt the estimated encroachment rate to capture the influence of road geometry on the maintenance cost of barriers. The adjusted maintenance cost of a barrier is computed employing Eqs. (6)–(9)

6 $${M}_{C}=\sum_{Y=a}^{a+l}\frac{{(1+i)}^{(Y-2023)}}{{(1+r)}^{(Y-2023)}} {(BMP}_{C}.L.{A}_{AADT}.{A}_{VM}.{A}_{enc})$$

7 $${A}_{AADT}=\frac{AADT}{Average AADT}$$

8 $${A}_{VM}=\frac{Vehicle Mix}{Average Vehicle Mix}$$

9 $${A}_{enc}=\frac{Modified Encroachment Rate}{Base Encroachment Rate}$$

where ${BMP}_{C}$ is the base maintenance cost of concrete barriers as listed in Table 4, which are the average values per foot per year based on data extracted from more than 150,000 work orders in California. The variables $a$ and $l$ are the start year and expected life of the barrier, respectively. ${A}_{AADT}$ is the modification factor based on the Annual Average Daily Traffic (AADT), ${A}_{VM}$ is the modification factor based on the vehicle mix, and ${A}_{enc}$ is modification factor based on the encroachment rate. The maintenance cost of steel barriers is computed similar to the concrete barriers.

The Eqs. (6)–(9) adjust the lifetime cost of each option based on economic factors (inflation rate and interest rate), AADT, vehicle mix, and crash rate. Adjusting the lifetime cost based on economic factors is standard for life cycle cost analysis studies as discussed in Section "LCC methodology". Additionally, adjusting the cost based on traffic volume, mix, and crash rate is pursued in the past LCC studies for vehicle crash costs. In this work we used these adjustment factors to modify the maintenance costs for the first time, as more car crashes directly translate to higher maintenance costs for barriers. The adjustment factors listed in Eqs. (7), (8), and (9) were introduced in several past studies, including the development of the RSAPv3 model and the Cooper model^5,7,20–22^.

Among the adjustment factors introduced in Eq. 6, ${A}_{enc}$ is the most complex and has been the subject of several studies^20,23,24^. Cooper et al.^20^ estimated the average encroachment rate using real-world crash and encroachment data collected from several Canadian provinces. They also presented a set of tables that modify the encroachment rate based on road geometry. The variables influencing encroachment include horizontal curvature, grade, number of lanes, lane width, shoulder width, terrain type, speed limit, and number of access points. The Cooper model provides independent tables to adjust the base encroachment rate based on these factors^20,23,25^. In this study, we employed the method by Cooper et al. to compute both the base encroachment rate and the modified encroachment rate listed in Eq. (9).

To account for the impact of traffic volume and traffic mix on maintenance costs, we introduced two other adjustment factors: ${A}_{VM}$ and ${A}_{AADT}$. Increased traffic volume leads to higher maintenance costs for barriers due to the increased frequency of crashes, including both major and minor incidents. To quantify this effect, ${A}_{AADT}$ is computed as the ratio of the traffic volume in the studied section of the road to the average traffic volume. The average traffic volume on highways was extracted from the Caltrans database for the years 2020 to 2022, providing a recent and relevant benchmark for our analysis.

Similarly, the traffic mix has a significant impact on maintenance costs. A crash involving a truck typically results in more substantial damage to roadside barriers compared to crashes involving smaller vehicles, leading to higher maintenance costs. We introduced ${A}_{VM}$ to adjust the maintenance cost based on the traffic mix. The average crash cost caused by different vehicle types—cars, pickup trucks, light-duty trucks, heavy-duty trucks, and motorcycles—is documented as part of the RSAPv3 model. In this study, we utilized the relative crash costs caused by each vehicle type to compute ${A}_{VM}$ similar to the method presented in RSAPv3^23^. The average traffic mix in California is comparable to the average traffic mix in the USA, as detailed by Nemati et al.^41^. Using this method, we computed the ${A}_{VM}$​ adjustment factor by calculating the weighted average of each vehicle category in the traffic mix reported in the studied section of the road. This approach ensures that the adjustment factors are based on the specific conditions of the study area.

### Equipment and workers exposure cost

In this study, four different categories of costs associated with exposure of workers and equipment to traffic during maintenance of barriers are considered:

o Worker fatality due to car crashes in the work zone.
o Worker injury due to car crashes in the work zone.
o Equipment damage due to car crashes during maintenance of barriers.
o Work injuries while performing the maintenance activity.

#### Workers’ fatality

A previous study conducted by the Advanced Highway Maintenance and Construction Technology (AHMCT) Research Center^26^ reported that the average fatal accident rate in California during maintenance activities from 1972 to 2013 was 2.18 × 10^–7^ deaths per person-hour of work on the road^27^. To compute the cost of worker fatality as a result of a car collision into the work zone, we used the most recent Valuation of a Statistical Life (VSL) published by the U.S. Department of Transportation^28,29^, which reports the VSL using the base year of 2022 as equal to $12.5 million^28^. Employing the VSL and average fatal accidents, we computed the cost of workers' fatality resulting from a car collision into the work zone to be equal to $2.57 per person-hour of work. Using the IMMS database, we extracted the yearly person-hours of work spent to maintain concrete barriers and steel guardrails. Caltrans has spent an average of 203,560 person-hours per year to maintain the barriers. Therefore, it can be estimated that the annual cost of workers' fatality due to exposure to high-speed traffic during maintenance of barriers is $523,149 per year.

#### Workers injury due to car collisions

Data from 2009 to 2022 provided by Caltrans indicated that there were 1,425 collisions in work zones that resulted in injuries to Caltrans employees, with the average cost of each injury equal to $28,459. A previous study^30^ reported that car collisions during the maintenance of barriers account for 3% of all Caltrans work zone collisions. Using this information, we computed that the cost of workers' exposure resulting from car collision injuries during the maintenance of the barriers is $0.43 per person-hour of work.

#### Equipment damage due to car collision

The total cost of accidents involving the Caltrans fleet amounted to $484,830 per year. This value includes all accidents that occurred while performing maintenance work on California’s highways and roads. Considering that car collisions during the maintenance of barriers make up 3% of all work zone car collisions, the cost of equipment damage due to exposure to high-speed traffic during the maintenance of barriers was estimated to be around $0.072 per hour of work.

#### Work injuries

We also computed the cost of workers' injuries while performing maintenance on concrete barriers and steel guardrails that are not the result of a car collision. Caltrans reported 313 incidents in the 2009 to 2022 period. A previous study reported that the average cost of work injuries was $8006 in 2006^31^. We adjusted that amount for inflation considering the change in VSL from 2006 to 2022^28^ and the average cost of work injuries in 2022 is estimated to be $15,745 per incident. Employing this information, we calculated the cost of work injuries to be equal to $1.73 per person-hour. As presented in Table 5, the total exposure cost is estimated to be $4.80 per person-hour of activity.

**Table 5:** The estimated cost of workers exposure during maintenance of barriers using Caltrans Motor Vehicles Incident database and Caltrans Personal Injuries database.

Description	Per person-hour
Fatality cost	$2.57
Injury due to car collision	$0.43
Equipment damage	$0.072
Work injuries	$1.73
**Total**	**$4.80**

By utilizing the data extracted from the Caltrans Motor Vehicles Incident database and the Caltrans Personal Injuries database, we computed the exposure cost for each type of barrier per foot per year. The results are listed in Table 6.

**Table 6:** Cost of exposure associated with each barrier type.

Barrier Type	Unit	Cost
Concrete Barrier	per foot per year	$0.334 × $${10}^{-2}$$
Thrie-Beam Barrier	per foot per year	$2.828 × $${10}^{-2}$$
W-Beam Barrier	per foot per year	$3.857 × $${10}^{-2}$$

The reported numbers in Table 6 include the sum of four categories of exposure cost. The frequency and total hours spent on maintenance activities on barriers depend on the collision rate and thereby road characteristics. In this work the exposure costs have been modified for the road characteristics, similar to the method used for maintenance cost adjustment presented in the previous section.

## Cost Imposed on Public

The choice of barrier influences the cost imposed on the public through traffic delays caused by lane closures during maintenance and repair of the barrier, as well as the change in probability and outcome of crashes with the barrier.

In prior cost–benefit analysis studies, installing a barrier is considered a benefit to the public, and this benefit is compared to the condition when the road has no barriers^4–6,21,32^. In this study, our baseline is not the road without a barrier, but rather we assume that some type of barrier must be installed on the roadside. Therefore, the costs imposed on the public through barrier choice are included.

### Crash outcome

The chance of a vehicle encroachment and crash into a barrier is significantly impacted by road geometry, speed limits, and weather conditions^33,34^. Many parameters such as horizontal curvature, vertical grade, lane width, shoulder width, and number of access points affect the probability of vehicle encroachment and crash into barriers^35^. Several studies have been performed estimating the encroachment and crash outcomes based on road geometry and conditions, and there are a few models available to compute crash costs. The three notable models and tools are the Roadside Safety Assessment Program (RSAPv3)^19,25^, Zhu model^24^, and Carrigan model^17^.

The Zhu model does not consider any differences in barrier types. The RSAPv3 model examines a particular segment of road and its roadside features to determine crash severity and cost. The Carrigan model can evaluate differences in crash probability and severity for general highways; however, it does not examine specific roadway geometry. Concrete barriers and steel guardrails exhibit different performance during car collisions, with passengers potentially sustaining more injuries when crashing into steel guardrails. For example, outcomes of the RSAPv3 showed that, on average, crashing into steel barriers results in a 23% higher crash cost than crashing into a concrete barrier for the vehicle.

Considering that RSAPv3 is the most comprehensive option among the available models and includes barrier type and road geometry to estimate crash cost, we utilized the RSAPv3 to calculate the crash cost associated with the barriers. Equation (10) presents the expected crash costs for segment *N* of the road.

10 $${E(CC)}_{N}=AADT\bullet {L}_{N}\bullet P(Encr)\bullet P(Cr|Encr)\bullet P(Se{v}_{s}|Cr)\bullet E({CC}_{s}|Se{v}_{s})$$

where $AADT$ is average daily traffic and ${L}_{N}$ is the length of segment $N$. The probabilities are represented as:

- ${\varvec{P}}({\varvec{E}}{\varvec{n}}{\varvec{c}}{\varvec{r}})$: The probability that a vehicle will encroach on the segment.
- ${\varvec{P}}({\varvec{C}}{\varvec{r}}|{\varvec{E}}{\varvec{n}}{\varvec{c}}{\varvec{r}})$: The probability of a crash if an encroachment has occurred.
- ${\varvec{P}}({\varvec{S}}{\varvec{e}}{{\varvec{v}}}_{{\varvec{s}}}|{\varvec{C}}{\varvec{r}})$: The probability of crash of severity, $s$, given a crash has occurred.

- ${\varvec{E}}({{\varvec{C}}{\varvec{C}}}_{{\varvec{s}}}|{\varvec{S}}{\varvec{e}}{{\varvec{v}}}_{{\varvec{s}}})$: The expected costs of a crash with severity.

RSAPv3 utilizes Cooper encroachment data to determine the model for computing the encroachment rate^20,23^. The Cooper data was collected from July to October 1978 by 12 teams from several Canadian provinces. The researchers monitored tire tracks and objects struck by vehicles on the roadside to determine the rate of encroachment^20^. The team that developed the RSAPv3 used this data to relate encroachment rate to road geometry and other factors.

One main drawback of the RSAPv3 is that it uses an iterative approach to compute the crash probability, which determines the crash cost. Therefore, executing the software takes a relatively long time. In this study, we employed a data-driven approach to estimate the RSAPv3 outcomes with low computation time for cases involving concrete and steel barriers as roadside features. We executed the RSAPv3 for various road geometries and conditions. In total, the probability, severity, and cost of a crash to concrete and steel barriers were computed for 232 situations using RSAPv3. Subsequently, we developed a regression model to regenerate the outcomes with adequate accuracy and low computational resources. Various models were tested to reproduce the RSAPv3 outcome with the least error. The regression model developed in this study to estimate the crash cost based on the computed encroachment probabilities is represented in Eq. (11).

11 $${C}_{s}={C}_{1}+{C}_{2}Gr+{C}_{3}{Gr}^{2}+{C}_{4}{Hz}^{-1}+{C}_{5}{Hz}^{-2}{+{C}_{6}S+{C}_{7}{S}^{2}+C}_{8}Enc+{C}_{9}{Enc}^{2}+{C}_{10}GrHz+{C}_{11}{Gr}^{2}Hz+{C}_{12}{GrHz}^{2}$$

**Figure 2:**
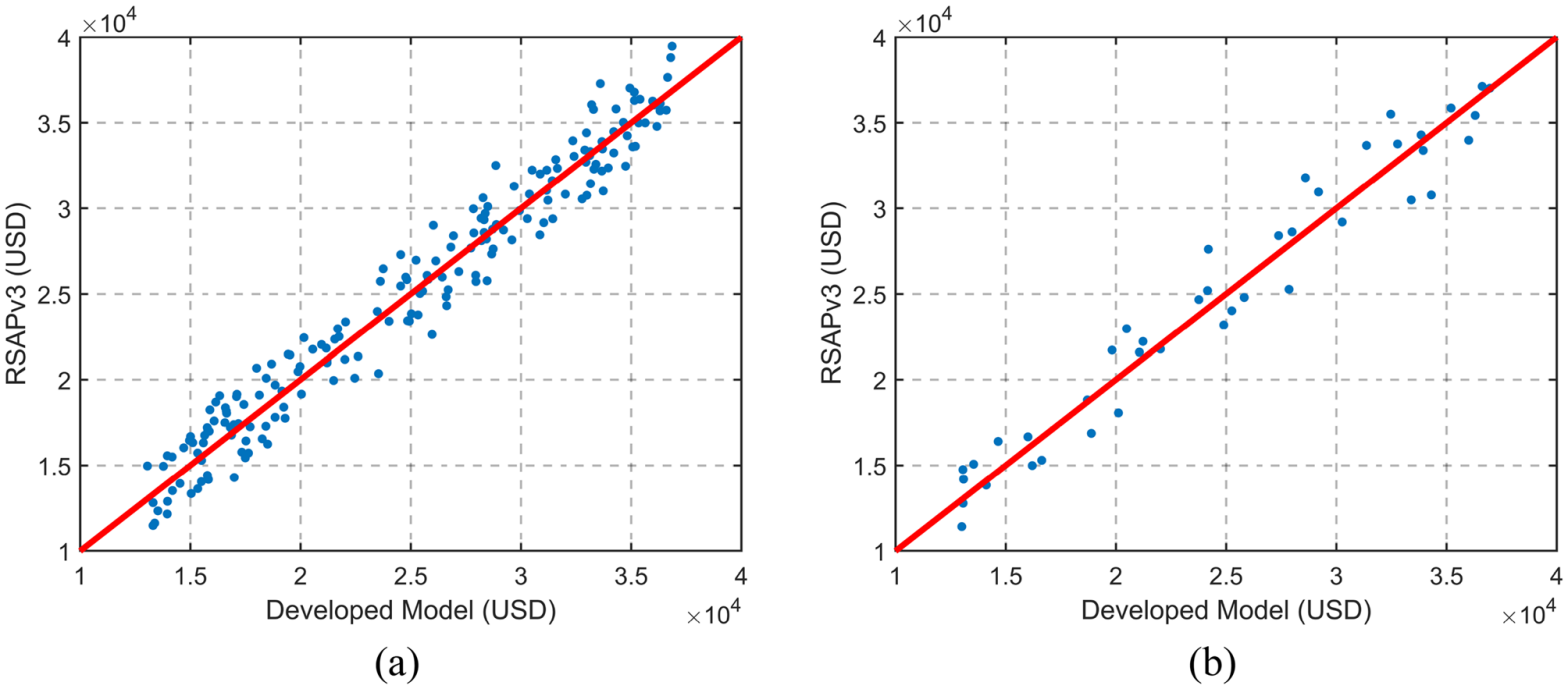
The estimated cost per encroachment computed by the RSAPv3 model and the developed model. (**a**) The model outcome during the parameter optimization, (**b**) the model outcome on test points.

In Eq. (11), ${C}_{s}$ is the crash cost of each encroachment, $Gr$ is vertical grade, $Hz$ is road curvature, $S$ is the posted speed limit, and $Enc$ is the encroachment rate computed using the Cooper model. The parameters ${C}_{1}$ to ${C}_{12}$ are listed in Table 7. Among the 232 data points, 192 points were used to optimize the model parameters, and 40 points were used to test the model. The test and training points were selected randomly. The ability of the model to reproduce the RSAPv3 outcome during model optimization and testing is illustrated in Fig. 2.

**Table 7:** Parameters for the regression model used to estimate crash cost adjusted for 2022.

Parameter	Value
C_1_	2.95 × ${10}^{5}$
C_2_	8.6010
C_3_	118.478
C_4_	− 42.366
C_5_	0.01084
C_6_	8867.96
C_7_	− 51.917
C_8_	− 297.811
C_9_	0.141962
C_10_	0.8951
C_11_	0.093611
C_12_	0.000322

As presented in Fig. 2b, the developed model is capable of reproducing crash costs to barriers on highways computed by RSAPv3 with adequate accuracy. The R-squared value of the outcome of the developed model compared to RSAPv3 is 0.967.

### Traffic delay

Several approaches have been introduced to compute the Value of Travel Time Saved (VTTS) for various road users, including leisure car drivers, business car drivers, truck drivers, etc.^13,36^. In this study, we used the most recent VTTS estimated and published by the U.S. Department of Transportation, which was reported to be equal to $20.17 per hour per vehicle in 2020^37^. We employed VTTS, the length of lane closure, time of lane closure, and AADT to compute the cost of travel delay resulting from lane closure due to maintenance activity. The cost of traffic delay was adjusted based on the geometry of the road. Table 8 lists the cost of traffic delay resulting from maintenance of barriers per foot per year per AADT. According to traffic rules, the speed limit reduces from 65 to 55 mph in construction zones. We computed the added travel time as a result of reduced traffic speed. It should be mentioned that occasionally lane closure due to maintenance activity results in a significant reduction of traffic speed. Some existing literature focuses on the estimation of reduced traffic speed based on the number of lanes, number of closed lanes, hourly traffic rate, and traffic mix^12,38^. Nevertheless, it is admitted that modeling traffic jams and accurate estimation of traffic speed reduction is non-trivial, and these studies only provide a rough estimation. It is noteworthy that the majority of the lane closures and work zones in highways in California do not cause speed reduction more than the posted speed limit^11,13^.

**Table 8:** Average estimated cost of traffic delay associated with each barrier type.

Barrier Type	Cost (USD/ft/year/AADT)	Duration of Lane Closure (hr/ft/year)	Length of Lane Closure (ft)
Concrete Barrier	$0.1101 × $${10}^{-6}$$	0.8367 × 10^–4^	5345
Thrie-Beam Barrier	$0.7234 × $${10}^{-6}$$	9.0101 × 10^–4^	3161
W-Beam Barrier	$4.298 × $${10}^{-6}$$	15.6141 × 10^–4^	11,184

Computing the traffic delay was conducted by extracting data from Caltrans IMMS database considering the average length of the work zone as well as the average duration of lane closure. The results presented in Table 8 show that concrete barriers require the least amount of lane closure per unit of length each year because they require less maintenance relative to steel barriers. However, if a lane closure is necessary to perform the repair, the length of lane closure is more than Thrie-beam steel barriers but shorter than the W-beam barriers. A previous AHMCT report showed that around 26% of maintenance activities on barriers require a lane closure^26^. We have added the traffic delay cost to the crash cost to compute the total cost imposed on the road users as follows:

12 $${P}_{s}={\sum }_{Y}\frac{{(1+i)}^{(Y-2023)}}{{(1+r)}^{(Y-2023)}}({C}_{s}.Enc.\frac{L}{5280}.\frac{VSL}{12.5}+{BTD}_{s}.AADT.L)$$

in Eq. (12), ${P}_{s}$ is the crash cost of the steel barrier, $Enc$ is the encroachment rate, $VSL$ is value of statistical life in million USD at the start of the project, ${BTD}_{s}$ is the base traffic delay cost presented in Table 8.

## The environmental considerations

In addition to the aforementioned factors, there are other environment related factors that influence the choice of barrier; however, they are difficult to quantify in terms of cost. This section provides an overview of some of those factors. The information presented in this section is gathered by extensive interviews with the maintenance and engineering personnel at Caltrans.

### Snow control and removal

It is among the key factors that impact the durability of roadside barriers. It is reported that some snowplow operators use the barriers as guides during snow removal operations, which can result in damage to the guardrails. Concrete barriers, on the other hand, are not typically damaged by snowplow machines, and only some visible wear marks without a need for repair are left on concrete barriers after a few years of installation. This concern has pushed some districts of California to replace steel guardrails with concrete barriers. Therefore, in areas where snowfall is regular, the impact of snow removal on the lifetime cost of barriers must be considered. In this work, this cost is accounted for through the reduced lifespan for the guardrails in the regions where frequent snow removal is needed.

### Water management

It was reported that there is not a significant cost difference between the two options when considering water management costs^39^. Small water passages can be included in the construction of concrete barriers without significantly affecting their construction cost, and only large drainage systems may add to the construction cost. In this work, we did not consider the water management cost as a separate factor due to the negligible difference between barrier choices. Nevertheless, in regions prone to flooding, steel guardrails are preferred over concrete barriers.

### Litter pick-up

The litter pick-up procedure is different around guardrails and concrete barriers. Sweeping machines are employed to clean roads with concrete barriers because of the risk of workers' exposure to high-speed traffic. However, litter pick-up around the guardrails can be done manually. It was found that quantifying the cost of litter pick-up is non-trivial due to the diversity in litter pick-up methods and different sources of labor during these operations. Thus, the difference in the cost of litter pick-up due to barrier choice is neglected in this study.

### Animal crossing

Large animals do not have a problem jumping over concrete barriers and steel guardrails^40^. For small animals, some openings are included in concrete barriers. It was explained during the interviews that the inclusion of the opening affects the construction costs minimally. On some occasions, large culverts are built to facilitate the crossing of animals and reduce accident risk. The procedure of building culverts is the same for steel guardrails and concrete barriers. Therefore, animal crossing measures do not create a noticeable cost difference between the considered barrier types.

## CalBarrier

Computing the lifetime cost of the barriers requires the inclusion of various parameters as presented in previous sections. To facilitate the comparison of the cost of concrete and steel barriers during their lifetime, we developed a software named CalBarrier. This tool was developed using MATLAB App Designer software (R2020a) and is structured as a single executable software. The design ensures straightforward usage and streamlines functionality. CalBarrier is optimized for operation on computers equipped with Windows operating systems, specifically Windows 7, 8, 10, and 11, and is freely available for public use^41^. A user manual and installation manual^41^ accompany the software are also available to public.

**Figure 3:**
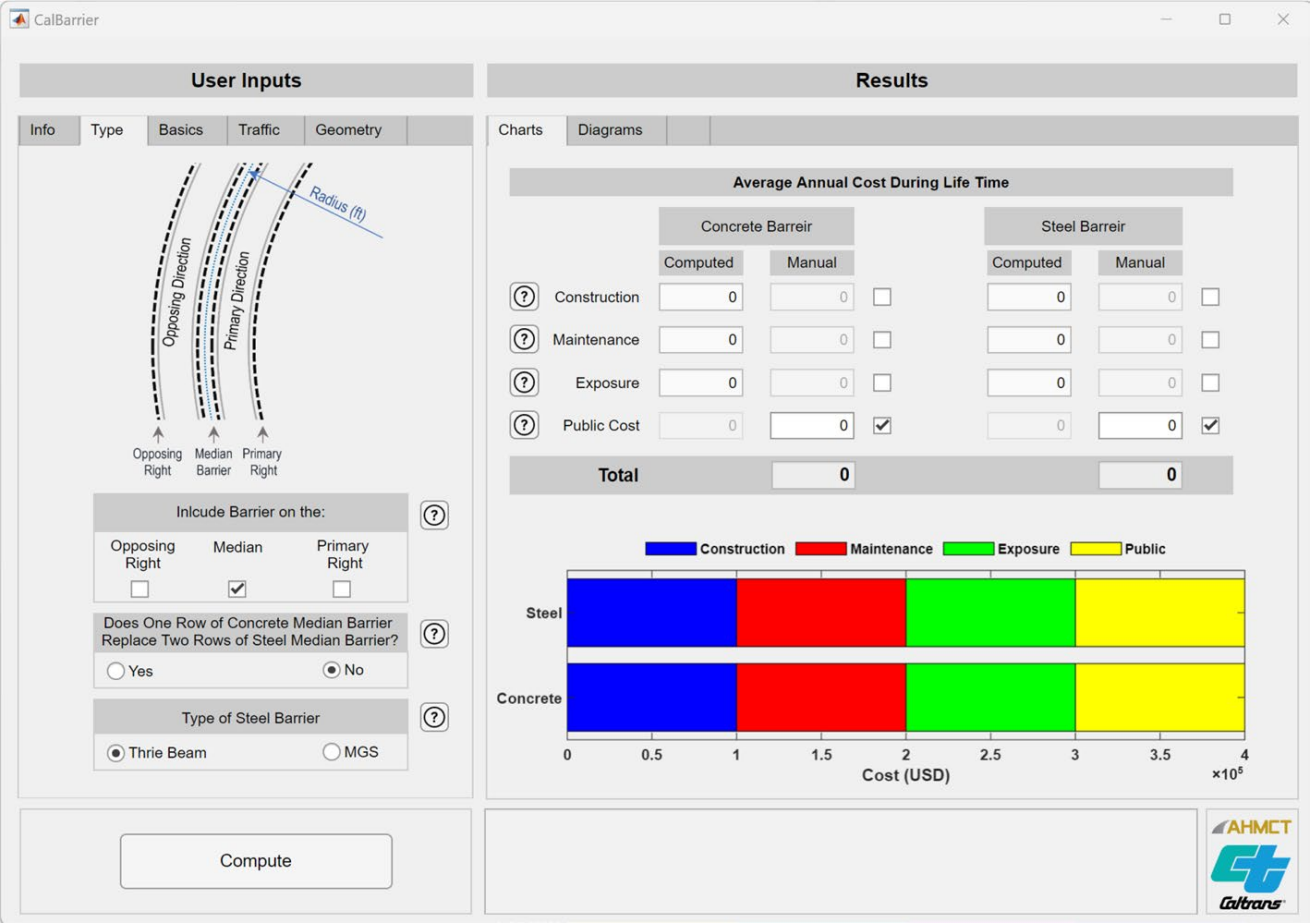
Snapshot of graphical user interface for CalBarrier^41^.

A snapshot of the software Graphical User Interface (GUI) for CalBarrier is shown in Fig. 3. The GUI encompasses two distinct sections: “User Inputs” and “Results”. On the left-hand side is the “User Inputs” section, which is comprised of different data entry tabs, each serving as a conduit for essential user-provided information. The “Results” section is on the right side of the GUI and consists of two tabs that present computed outcomes through charts and diagrams.

The “User Inputs” segment is divided into five tabs: “Info”, “Type”, “Basics”, “Traffic”, and “Geometry”. As shown in Fig. 3, the “Type” tab has been activated.

Within the “Results” section, users are presented with two tabs: “Charts” and “Diagrams”. In the “Charts” tab, the average annual cost of concrete and steel barriers is detailed. In the “Diagrams” tab, users can readily grasp the temporal distribution of costs, as well as the relative impact of various cost factors on the lifetime cost of each barrier.

### ***Examples***

In this section, two examples of calculating lifetime of different barrier options are presented. These examples demonstrate the significant impact of various factors on the total lifetime cost of barriers. A comprehensive sensitivity analysis to quantify the impact of different inputs on the life cycle cost of barriers is left for a future study. Table 9 lists all the parameters used in the two examples. The costs presented in Table 9 are annual average cost, which means the initial costs are divided over the lifetime cost of the barriers. The yearly occurring costs are also adjusted to presented value as described earlier in Section "LCC Methodology".

**Table 9:** Two examples of computing the lifetime cost of concrete and steel barriers.

Common factors						
Barrier Location	*Median*		Steel Barrier Type		*Thrie-Beam*	
Regular Snow removal	*No*		Number of Lane		*4*	
Inflation Rate	*2.2%*		Lane Width		*12 ft*	
Interest Rate	*4.2%*		Access Density		*0*	
Expected Life, Concrete	*50 years*		Rumble Strip		*Present*	
Expected Life, Steel	*50 years*		Terrain Type		*Flat*	
AADT	*100,000*		Speed Limit		*65 mph*	
Average growth of AADT	*0%*		Length of Road Section		*10,000 ft*	
Traffic Mix	*Car (68%), Pickup (20%), Light Truck (6%), Heavy Truck (4%), Motorcycle (2%)*					

	Example 1			Example 2		
Vegetation Management	*Not Necessary*			*Necessary*		
Horizontal Radius	*Straight*			*1200 ft*		
Grade	*0*			*1%*		
Shared Median	*No*			*Yes*		

	Results (Average Annual Cost)					
	Concrete	Steel		Concrete	Steel	
Construction Cost	*$42,981*	*$18,134*		*$21,491*	*$26,437*	
Maintenance Cost	*$921*	*$12,408*		*$1,420*	*$19,126*	
Exposure Cost	*$41*	*$347*		*$63*	*$535*	
**Ownership Cost**	**$43,943**	**$30,889**		**$22,974**	**$46,098**	
Public Cost	*$190,225*	*$234,737*		*$407,461*	*$501,938*	
**Total Cost**	**$234,168**	**$265,626**		**$430,435**	**$548,036**	

Many factors are similar in the two examples. The inflation and interest rate, the expected lifetime, number and width of lanes, shoulder width, speed limit, traffic volume and mix, terrain type, and presence of rumble strip are among the factors that are the same for both examples presented in Table 9. In example 1, the road is straight, has not vertical grade, there is no need for extra vegetation control and the median barrier is not shared for the two directions of the road. On the other hand, in example 2, road is curved to the right with radius of 1200 ft measured at the road centerline, it has vertical grade of 1% (uphill), vegetation management is necessary, and one row of concrete median barrier is enough for both direction of the road. As the results presented in Table 9 illustrates, maintenance costs and, accordingly, the exposure of workers and equipment are higher in example 2 compared to example 1 as the horizontal curvature and grade increase the probability of encroachment and crashes into the barrier. In example 2, the maintenance cost is substantial, resulting in increased lifetime ownership cost of steel barriers. Additionally, the additional requirement to prevent vegetation growth around the steel barriers increases their initial construction cost.

It is common to install only one row of median concrete barrier when the median is not very wide. This practice substantially reduces the initial cost of the median concrete barriers as in example 2. The inability to have shared barrier and extra requirement for vegetation management may cause the steel barriers to have a higher initial cost (as presented in example 2) compared with concrete barriers. Nevertheless, the example 1 demonstrates that in straight sections of roads in areas with no additional requirement for vegetation management, steel barriers are significantly cheaper and have lower lifetime costs compared to concrete barriers.

Concrete barriers have been shown to be less dangerous to vehicle occupants during crashes compared to steel barriers. Considering the public cost shows that concrete barriers are cheaper during their lifetime compared to their steel counterparts. However, the public cost is not usually an out-of-pocket cost for many organizations and agencies. Therefore, it is a common practice not to include the public cost in the computation of the lifetime cost of barriers with the same weight as initial and maintenance costs.

## Conclusion

In this study, we investigated the life cycle costs associated with concrete and two types of steel barriers. We incorporated factors such as initial costs, maintenance expenses, worker and equipment exposure during maintenance, and the indirect costs imposed on the public. We conducted interviews with personnel from the California Department of Transportation to gather informationn on the critical factors impacting the cost of barriers. We adjusted the lifetime cost of each barrier based on the road geometry, traffic volume and mix, weather conditions, and economic factors, including inflation and interest rates. Raw data from over 150,000 job orders in California from 2020 and 2022 period were extracted from various Caltrans databases to calculate the construction, maintenance, and exposure costs associated with each barrier type. The Cooper model and RSAPv3 model were employed to compute the encroachment rate and crash cost, respectively. Correlating the frequency of barrier maintenance with their encroachment rate, we modified the maintenance and exposure costs based on their location-dependant encroachment rate. Furthermore, we developed a data-driven model to estimate the crash cost from RSAPv3 at low cost.

The software tool, CalBarrier, developed as part of this study, embodies our methodology and makes it accessible for practical application. CalBarrier allows users to input region-specific data to compute and compare the lifetime costs of different barrier types, ensuring the transferability of our findings to various regions and conditions.

While our study focuses on data from California, the methodology and software are designed to be applicable globally, making adjustments for local conditions and costs straightforward.

We anticipate that the findings from this study will be directly relevant to areas sharing socioeconomic characteristics with California, USA and to other regions by adjusting the user input base costs for each category.

Future research will include a comprehensive sensitivity analysis to further validate the robustness of our equations and the influence of various input parameters on the lifetime costs of barriers. In addition, to account for additional environmental effects such as increased vehicle emissions due to barrier maintenance and repair, and finally, deploying the developed methods and computed cost in this work for production of tools that can be used for optimal maintenance scheduling.

## Data Availability

The processed data that support the findings of this study are available on request from the corresponding author, Shima Nazari. The raw data are not publicly available due to restrictions implemented by California Department of Transportation.
